# From calls to action: leveraging the 2-1-1 system to intervene on food insecurity

**DOI:** 10.1093/haschl/qxag063

**Published:** 2026-03-14

**Authors:** Nasser Sharareh, Gloria Castaneda, Rachel Dalrymple, Cassandra Wambach, Ben Brintz, Sara E Simonsen, Olutobi A Sanuade, Hanna Hedges, Fernando A Wilson, Rachel Hess, Jorie Butler, Andrea S Wallace

**Affiliations:** Department of Population Health Sciences, Spencer Fox Eccles School of Medicine at the University of Utah, Salt Lake City, UT 84108, USA; Utah's Promise, United Way of Salt Lake, Salt Lake City, UT 84108, USA; Department of Biomedical Informatics, Spencer Fox Eccles School of Medicine at the University of Utah, Salt Lake City, UT 84108, USA; Utah's Promise, United Way of Salt Lake, Salt Lake City, UT 84108, USA; Department of Internal Medicine, Spencer Fox Eccles School of Medicine at the University of Utah, Salt Lake City, UT 84108, USA; College of Nursing, University of Utah, Salt Lake City, UT 84108, USA; Department of Population Health Sciences, Spencer Fox Eccles School of Medicine at the University of Utah, Salt Lake City, UT 84108, USA; Clinical and Translational Science Institute, University of Utah, Salt Lake City, UT 84108, USA; Department of Population Health Sciences, Spencer Fox Eccles School of Medicine at the University of Utah, Salt Lake City, UT 84108, USA; Department of Economics, College of Social and Behavioral Science, University of Utah, Salt Lake City, UT 84108, USA; Matheson Center for Health Care Studies, University of Utah, Salt Lake City, UT 84108, USA; Department of Population Health Sciences, Spencer Fox Eccles School of Medicine at the University of Utah, Salt Lake City, UT 84108, USA; Department of Biomedical Informatics, Spencer Fox Eccles School of Medicine at the University of Utah, Salt Lake City, UT 84108, USA; College of Nursing, University of Utah, Salt Lake City, UT 84108, USA

**Keywords:** public health, surveillance systems, nutritional education, information, SNAP

## Abstract

**Introduction:**

The 2-1-1 system is a nationwide social service provider that connects callers with unmet needs to appropriate community resources. Every year, over two million Americans experiencing food insecurity (FI) seek help from 2-1-1. Yet, little is known about the FI experiences among 2-1-1 callers seeking food-related support, specifically, who they are, the circumstances that lead them to contact 2-1-1, and the strategies they believe could help reduce FI.

**Methods:**

To address this gap, we interviewed 30 food-related callers (20 in English and 10 in Spanish) to the Utah 2-1-1 system between November 2024 and January 2025.

**Results:**

Thematic analysis revealed three primary reasons for seeking food-related help from 2-1-1: (1) ineligibility for federal nutrition and financial assistance programs; (2) facing non-income-related barriers to accessing food; and (3) experiencing unexpected life events. Callers suggested strengthening and adapting federal nutrition assistance programs, improving emergency food provision systems, and enhancing informational support.

**Conclusion:**

Based on these insights, we provide suggestions on how to leverage the 2-1-1 system to intervene on FI.

## Introduction

People with unmet food needs use different ways to find help with food: some people rely on their existing information to access food assistance programs, some reach out to their social and healthcare network, and some will reach out to social service providers. One of the major social service providers is the 2-1-1 network—a non-profit, free referral system and the most comprehensive source of information about local resources in the United States (U.S.)^1^ Analogous to 911, 2-1-1 is a telephone number that provides information and referrals to social assistance programs. There are more than 200 2-1-1 agencies across the U.S.^1^ They all provide services in 180 languages using non-English service navigators or a translation line. At the time of an incoming call, a 2-1-1 service navigator documents the caller's ZIP Code, biological sex, primary language, time, and reason for the call, which is identified using a standard taxonomy. A call is flagged as food-related if there is any indication of unmet food needs or asking for help with food. In 2024 alone, 2-1-1 made 2.5 million connections to food resources, with food-related calls being the third most common reason for 2-1-1 calls nationwide, after housing and utilities.^2^

Prior research has used 2-1-1 food-related call volumes to identify geographic service gaps and to inform resource allocation,^3,4^ such as placing an emergency food asset in a ZIP Code with high food-related call volume but no food pantries nearby.^3^ Analysis of transcripts of 2-1-1 food-related calls has shown evidence of food insecurity (**FI**), defined as limited access to adequate food for an active, healthy life, among callers.^5^ Specifically, callers often mentioned a lack of money to afford food or having other FI-related risk factors, such as transportation barriers and unemployment at the time of the call.^5^ However, there remains a lack of in-depth analysis of FI experiences among 2-1-1 food-related callers, particularly in understanding who these individuals are and the circumstances that led them to contact 2-1-1. Also, most 2-1-1 food-related callers are referred to emergency food providers, such as food pantries, to address their immediate food needs. However, less is known about other ways this system could be used to intervene on FI. To the best of our knowledge, prior research has not explored suggestions that the 2-1-1 food-related callers may have on strategies to reduce their FI. This is an important missed opportunity for at least three reasons: (1) 2-1-1 is available nationwide and any interventions, either implemented through or informed by this system, could have a significant impact; (2) although 2-1-1 food-related callers may not represent all individuals at risk for or experiencing FI, they tend to over-represent populations facing health disparities, including racial, ethnic minority groups and low-income Americans,^6^ who also have the highest rates of FI in the U.S.;^7,8^ and (3) 2-1-1 captures calls from individuals across various income levels; calls come not just from people in or near poverty, but also from those whose income exceeds the poverty threshold and are newly in need.^9^ In fact, during the pandemic, a higher proportion of food-related calls came from mid-poverty ZIP Codes (ZIP Codes where 5% to 25% of the population live in poverty.)^10,11^ This is important because two-thirds of households with FI have incomes above the poverty line.^7,8^

While other social service providers may offer similar support, 2-1-1 stands out for its national reach—serving approximately 99% of the U.S. population—^2,12^ and its ability to share call volume data with researchers and policymakers within a day. Therefore, this timely research can inform how to leverage the 2-1-1 system to intervene on FI by exploring the FI experiences of 2-1-1 food-related callers and their suggested strategies to address FI.

## Data and methods

This was a qualitative study involving semi-structured interviews with food-related callers to Utah's 2-1-1 system. The University of Utah Institutional Review Board approved this project (IRB_00176706). Methods for data collection, analysis, and reporting were guided by the Consolidated Criteria for Reporting Qualitative Research (COREQ) guidelines (see Appendix, Table S1 for details).

### Recruitment

From November, 21, 2024 to January, 23, 2025, United Way of Salt Lake (**UWSL**), the operator of 2-1-1 in Utah, identified 55 2-1-1 food-related callers (36 English speaking and 19 Spanish speaking) who met the inclusion criteria of being an adult aged 18 years or older and having food-related needs, and who were interested in participating in a research study related to FI. The UWSL shared the contact info of those 55 callers with our research team. The coordinators called all 55 callers to invite them to participate and schedule interviews. Three callers declined participation. Eleven callers could not be reached, despite three contact attempts and a final follow-up text message. Eleven callers were busy at the time of contact and did not respond to subsequent follow-ups scheduled at mutually agreed-upon times. Thirty callers were ultimately interviewed, 20 in English and 10 in Spanish.

### Data collection

The Community Collaboration and Engagement Team (CCET) at the Utah Clinical and Translational Science Institute conducted all 30 semi-structured interviews. Semi-structured interviews allow participants to elaborate on their responses beyond the predetermined questions.^13^ CCET staff were trained on Human Subject Research and conducting interviews. All interviews were conducted remotely and were audio-recorded with the participant's permission. Most interviews lasted an hour. Interviews were conducted in either English or Spanish based on participants' language preferences and to ensure inclusivity in data collection. Participants were paid $50 for their time. Before the interview, we provided participants with a written consent cover letter, and participants provided oral consent, which was recorded and transcribed.

Before the interview, we identified participants' demographic information and assessed their FI and nutrition insecurity status, use of nutrition assistance programs, FI-related risk factors, and diet-related health conditions. FI risk was assessed using the two-item Hunger Vital Sign screener:^14^ (1) “Within the past 12 months, we worried whether our food would run out before we got money to buy more,” and (2) “Within the past 12 months, the food we bought just didn’t last and we didn’t have money to get more.” Response options included often true, sometimes true, or never true. Participants who answered “often true” or “sometimes true” to either question were considered at risk for FI. For nutrition insecurity, we used a 1-item screener developed by the Center for Nutrition & Health Impact^15^: “In the last 12 months, we worried that the food we were able to eat would hurt our health and well-being.” Response options included never, rarely, sometimes, often, always, and don’t know. If participants answered “often” or “always”, they were considered to have nutrition insecurity. For access to Supplemental Nutrition Assistance Program (SNAP), we asked: “At any time in the last 12 months (and last month), did you or any family members receive SNAP (food stamp) benefits?” (Yes/No). For emergency food providers' utilization, we asked: “At any time in the last 12 months (and last month), did you or any family members ever get free groceries or meals from a food pantry, food bank, church, or other place that helps with free food and meals?” (Yes/No). To assess FI-related risk factors, we asked: (1) “In the past 12 months, have you had trouble paying for your utilities”; (2) “Are you worried or concerned that in the next two months, you may not have stable housing that you own, rent, or stay in”; and (3) “In the past 12 months, has lack of transportation kept you from medical appointments, meetings, work, or getting help with food”. To assess diet-related health conditions, we used screener questions from the National Health Interview Survey: “Has a doctor ever told you that you had type 2 diabetes or hypertension or coronary heart disease?” (Yes/No).

Throughout the interview, we used open-ended questions to explore FI experiences among participants, focused specifically on identifying details related to the reasons for, and expectations of, the call: (1) “You recently called 2-1-1 because you needed help with food; 2) can you please explain what happened that day or week that made you call 2-1-1 to seek help for food?” and (3) “What kind of support or information were you hoping to get from 2-1-1 when you called them?”. Additionally, two questions sought callers' preferred strategies for addressing their food needs: (1) “What do you think is needed in your neighborhood to improve your access to food?” and (2) “Do you have any suggestions for how 2-1-1 could better support people when they need help with food?”.

### Data analysis

The responses to demographic and food-related questionnaires were summarized and reported as percentages. Interview audio recordings were translated to English, as required, transcribed, and entered into qualitative software for analysis (Dedoose, Version 9.2.012). The qualitative analysis team consisted of three researchers with expertise in FI, qualitative analysis, and community-engaged research (NS, RB, JMB; one male and two females). We used Braun and Clarke's thematic analysis approach to analyze the transcripts. Thematic analysis organizes qualitative data into codes and themes similar to other conventional qualitative methods (eg, grounded theory, ethnography) and works well for exploring experiences among research participants.^16,17^ This method also emphasizes researchers' active role in theme development, the iterative and interpretive nature of coding, and the generation of themes as patterns of shared meaning rather than quantifiable categories. First, a codebook was developed based on the first two transcripts. Then, RB and NS coded the remaining transcripts according to the agreement in the group coding session. We then resolved disagreements in code names or coded quotes through discussion and coder consensus. Similar codes were grouped together to represent preliminary themes. We then continued to modify themes until the final themes were accurate and meaningful and did not overlap with each other. The process revealed that thematic saturation^18,19^ (ie, the point at which transcripts do not reveal new information) was reached and, thus, no additional interviews were sought.

### Limitations

This study has a few limitations. We interviewed only English and Spanish-speaking callers, and most participants lived in urban areas of Utah, which may have different resources and services than other regions of the country. In addition, some individuals may use the 2-1-1 website instead of calling 2-1-1 to find help with food. Therefore, future research should explore whether the FI experiences and suggested strategies of callers speaking other languages, rely on the 2-1-1 website, or live in rural areas align with our findings. Because we only interviewed 30 callers, we cannot speak to the generalizability of our findings. Nevertheless, findings offer valuable insights into the lived experiences of individuals at risk for FI who seek assistance and may inform solutions to consider in future interventions. We also contend that the strategies proposed to address FI, along with the experiences shared by these callers, should not be viewed as isolated events. Prior research indicates that people experiencing FI often report similar patterns and challenges, which may reflect the structural barriers in accessing food in the U.S. At the same time, we acknowledge that some contextual features of Utah may influence the experiences of callers in ways that differ from other regions. For example, Utah's restrictions on completing SNAP applications through 2-1-1, the specific configuration of local food pantry networks, and the particular distribution of urban vs rural service areas may shape callers' access pathways differently than in other states.

## Results

### Callers' characteristics

The characteristics of participants are described in Table 1. Thirty callers were interviewed, 20 in English and 10 in Spanish. Participants had an average age of 47, 33% were female, 47% were married, 40% were non-Hispanic White and 47% were Hispanic, 60% had a high school degree or below, 37% were employed either part-time or full-time, 60% had health insurance, 37% had a disability, 80% had access to a private vehicle, 90% had access to internet, and 100% had access to a phone. Participants' household size was on average 3.5, with 87% of participants having children in their households. The majority of participants (87%) had incomes below 185% of the federal poverty level, and 63% had incomes below 130% of the federal poverty level, which is the income threshold for SNAP eligibility in Utah.

**Table 1.** Characteristics of 30 interviewed 2-1-1 food-related callers.

Variable	% (N)
**Language of Interview**	
** English**	67% (20)
** Spanish**	33% (10)
**Average age**	47 (Min: 20—Max: 76)
**Sex**	
** Female**	33% (10)
** Male**	67% (20)
**Married**	47% (14)
**Race/Ethnicity**	
** Non-Hispanic White**	40% (12)
** Non-Hispanic Black or African American**	7% (2)
** Native Hawaiian**	3% (1)
** Asian**	3% (1)
** Hispanic**	47% (14)
**Education**	
** High school degree or below**	60% (18)
** Some college or above**	40% (12)
**Employed (part-time or full-time)**	37% (11)
**Average household size**	Average: 3.5 (Min 1—Max 7)
**Children in the household**	87% (26)
**Children under 18 in the household**	50% (15)
**Children under 5 in the household**	40% (12)
**Income less than 185% of the federal poverty level**	87% (26)
**Income less than 130% of the federal poverty level**	63% (19)
**Insured**	60% (18)
**Have a disability**	37% (11)
**Access to a private vehicle**	80% (24)
**Access to the internet**	90% (27)
**Access to a phone**	100% (30)
**Transportation barriers**	43% (13)
**Difficulties paying for utilities**	87% (26)
**Housing insecurity**	60% (18)
**At risk for food insecurity in the past 12 months**	97% (29)
**Nutrition insecurity in the past 12 months**	60% (18)
**SNAP utilization in the past 12 months**	30% (9)
**SNAP utilization in the past month**	30% (9)
**Food pantry and other free food resources (eg, church) utilization in the past 12 months**	63% (19)
**Food pantry and other free food resources (eg, church) utilization in the past month**	53% (16)
**Diabetes**	23% (7)
**Heart disease**	23% (7)
**Hypertension**	33% (10)
**Obesity**	30% (9)

Per the standardized questionnaires, 97% of participants were at risk for FI, and 60% had nutrition insecurity. Many participants had not been previously connected to food programs. Less than one-third (30%) used SNAP, and less than two-thirds (63%) used food pantries in the past 12 months. Among those not enrolled in SNAP, 60% were potentially eligible based on their reported annual income.

FI-related risk factors were also common among participants: 47% reported transportation barriers, 60% reported housing insecurity, 87% reported difficulties paying household utilities.

Finally, diet-related health conditions were prevalent in this sample: 23% had diabetes, 23% had heart disease, 33% had hypertension, and 30% had obesity.

### Qualitative findings

After exploring the reasons for calling 2-1-1, three themes emerged: Ineligibility for federal nutrition and financial assistance programs, non-income-related barriers to accessing food, and unexpected life events experiences.

#### Ineligibility for federal nutrition and financial assistance programs

Many callers were ineligible for SNAP or other federal financial support due to exceeding the necessary income requirements or immigration and citizenship status. See Table 2 for relevant quotes.

**Table 2.** Reasons why people with unmet food needs call 2-1-1.

Theme	Relevant quote followed by participant ID, sex, the interview language, family composition, and age.
**Ineligibility for federal nutrition and financial assistance programs**	Well, I applied a while ago to workforce services over food a couple times, but **they say we made too much**, which we made, but all the money goes out to other stuff. She worked at [City Name], she spends a lot of money on gas in [City Name] back and forth, and it just doesn’t last, money doesn’t last.” (ID 14, Male, English, Married, Has kids, 65) Even if they’re working, making $20, $21 an hour, it's hard for you to get by because if you have kids and you got your rent and your car note and insurance and all other bills stuff going on, by the time you get your check, the whole check is gone. It comes and goes straight to the bills, and they make that program so hard. So if you’re working, and like I said, it goes by how big your family is. So a family of six, they say you can’t make more than $3,000, but then **they’re adding up the gross, not the take-home money**. They make it so hard for people to get help. Mostly everything is so expensive, the food is so expensive. I think if they changed the way their system was and just help American people, that would be great.” (ID 10, Male, English, Married, Has Kids, 40) So yes, it was an agent at Rocky Mountain Power that gave me the places to call for the heat assistance and those kinds of things, but we barely don’t qualify, so we make just barely enough over the minimum so **we don’t qualify for any heat assistance.** So it really was not successful.” (ID 10, Male, English, Married, Has Kids, 40) No, I never received any food stamp benefit. I did try to go get it, but again, **because of my status, they said I cannot get it**. I don’t have a social security number.” (ID 22, Female, English, Divorced, Has kids, 45) Oh, the food stamps, miss? Because I am still in the immigration process. As a newcomer, **I was told I couldn’t apply for that** because it's only for residents.” (ID 26, Female, Spanish, Single, No kids, 31)
**Non-income-related barriers to accessing food**	**I don’t see anything my kids or me like**. You can’t eat it because you have pork and chicken, but I don’t eat… Just halal, some chicken or beef halal.” (ID 3, Female, English, Married, Has Kids, 36). You can just tell by the way they talk to you sometimes or how they treat you, you’re below them. I mean, sometimes **if someone's been discriminated against or if somebody's felt like they were less than, sometimes people just don’t go back because they feel like they’re not worthy to go back**.” (ID 5, Female, English, Single, Has Kids, 34) **I have had double vision and migraines**. I have been going to an eye doctor and a neurologist trying to take care of those. I’ve had to have somebody drive me a lot.” (ID 1, Female, English, Divorced, Has kids, 65) One family member, she lives in [City Name]. Well, I guess there's very limited and good pantries over in her area to go to and **she don’t have a car, so I don’t know how she does it**. She can’t drive. She's so disabled that she's not allowed for a driver's license to drive any longer.” (ID 13 Female, English, Single, Has kids, 41) I have not gone to the food pantries because **my ego will not let me get there** yet, as stupid as that is. So that's tough to answer I guess.” (ID 4, Female, English, Married, Has kids, 52)
**Unexpected life events experiences**	Yes, what happened is that last year in April, our three-year-old daughter was **diagnosed with cancer.** Because of this, I had to **quit my job**, I was **pregnant.** And that's when everything started to go downhill. We focused on her recovery, her treatment, being attentive to her. My **husband started working less** too. And that led us to a financial crisis. Thank God she is now cured of cancer. But now we are practically in debt.” (ID 23, Female, Spanish, Married, Has kids, 36) I just have so many bills and don’t make enough to actually pay for my food. Money has been tight for me my whole life. It's never not been. Yeah, it is just never not been. And I am alone. It's just me. So no matter how many jobs I work, it has never been enough to pay off my **student loans.** I had to have **surgery**. My **car stopped working** earlier this year and I had to get a new one. And then I was **homeless,** and I had to put rent on a credit card and **haven’t been able to pay off the credit card**. And it's just years of not quite having enough has come all on me at once.” (ID 9, Female, English, Divorced, No kids, 29) Can’t really say that I foresee what else is going to happen because **we really haven’t been here** yet in this situation before to even know.” (ID 4 Female, English, Married, Has kids, 52) **I’ve never been in this situation** ever in my life and it's scary and unforeseen.” (ID 29, Male, English, Single, Has kids, 46) Honestly, **I don’t know what benefits or resources I can get**. I found out about 211 out of desperation, searching on the internet, and it said we could call 211 for help, so I called (ID 25, Female, Spanish, Married, Has kids, 21)

#### Non-income-related barriers to accessing food

Many callers described that they had experienced a variety of barriers that had prevented them from accessing support for food, including not finding culturally appropriate foods, feeling discriminated against, experiencing health issues, facing transportation barriers, experiencing personal and societal stigma, and not knowing how to address their unmet food needs.

#### Unexpected life events experiences

Many individuals calling 2-1-1 had experienced a variety of factors that led to FI. One caller described how the combination of a health diagnosis, pregnancy, and job loss led to a financial crisis. Another caller described how debt, car issues, surgery, and homelessness made it difficult to find the money for food. One cohort of individuals calling 2-1-1 had no previous experience with FI and were unfamiliar with how to address the issue. One caller explained they did not know how their situation would resolve or progress. Another caller expressed their fear in navigating FI, due to it being a new situation for them (see Table 2).

Caller's suggested strategies to address FI also included three themes: Strengthen and adapt federal nutrition assistance programs, improve the emergency food provision system, and enhance informational support.

#### Strengthen and adapt federal nutrition assistance programs

Some callers suggested revising eligibility rules to better serve those with temporary needs and increasing benefit amounts to reflect inflation (see Table 3).

**Table 3.** Suggested strategies to reduce food insecurity by 2-1-1 food-related callers.

Theme	Relevant quote followed by participant ID, sex, the interview language, family composition, and age.
**Strengthen and adapt federal nutrition assistance programs**	I think there should be **a different criterion when qualifying people** to see if they qualify or not. Because there could be programs that can help. There are people who, let's say, for example, middle-class people who need help, for example, with food or paying a bill, they can get temporary help. It doesn’t mean they need help forever. No, but since there are many people who hang on to the system, so to speak, they live off the system, asking for help month after month, it makes it difficult for people who need help only for, let's say, a month or two. I explain, temporary. So, ah. The people who really need help at the moment don’t get it because the help is being given to those who live off the help, so to speak, forever, so to speak. Or it's something like a routine. So, I think there should be a system that if you need help, let's say for a month or two, you get the help, you get out, and you move on. Right?” (ID 15, Female, Spanish, Married, Has kids, 36) With SNAP, I think they should take into consideration that inflation and the cost of food have gone up, so maybe **they should reevaluate the amounts that they’re giving**.” (ID 12, Female, English, Single, Has kids, 54)
**Improve emergency food provision system**	I just think that if they would **allow people to go more often,** it wouldn’t be as big of an issue, especially when you have a larger household.” (ID 6, Female, English, Single, No kids, 28) I’m so scared of those food pantries and after going the last time and all I got was **expired food**. I don’t have the time or the energy to go and get expired food. I’d rather just dig up what money I can or whatever and go buy ramen noodles” (ID 1, Female, English, Divorced, Has kids, 65) I go for some place. But I go for late and I don’t see anything my kids or me like. You can’t eat it because it is pork and chicken, but I don’t eat… Just halal some chicken or beef halal” (ID 3, Female, English, Married, Has kids, 36) Maybe they can, what they’re giving out for food, they can **make sure each box has meal stuff you think would go together for a meal** in it and remember every ingredient that would help to make different types of meals with those ingredients.” (ID 13, Female, English, Single, Has kids, 41) I think a **mobile food truck** will be great for people that don’t have any type of transportation to get to. Because if you live here in [City Name] and there's no food bank around here, there's no food pantries around here. So you got to go down to [City Name] to get the food banks or downtown. So if you don’t have a car then you won’t be able to make it down there. So I think that will be **helpful for people that don’t have a car.” (**ID 10, Male, English, Married, Has kids, 40) They (food pantries) weren’t that bad. They were real friendly. They treat everybody the same. **They’re just not available all the time.” (**ID 11, Male, English, Single, Has kids, 41) They need to have more food banks open every day instead of just three days and only for certain hours. If you don’t have a car, how are you going to get to the food bank? They need to have ways to get people to these food banks and help them carry their food up to their place where they live (ID 18, Female, English, Married, No kids, 66)
**Enhance informational support**	I **didn’t know how to check what they gave me**. Because when I told my daughter, she said no, no, no. She didn’t have time. So, I didn’t check anything. Honestly, I didn’t check because **I don’t know much about emails.**” (ID 8, Female, Spanish, Widowed, Has kids, 73) **Sometimes we don’t get notified all the time**. And I know that my life is really hectic sometimes, and so I don’t remember every single day that I have something scheduled, let alone a phone interview or an application thing online, you know?” (ID 5, Female, English, Single, Has kids, 34) Well, I think more than anything, **communication with the Hispanic community,** having **meetings to make it known** because, look, I honestly didn’t know they gave coupons, that the university cares about the Hispanic community. But many, **many unfortunately don’t know**. And honestly, it's good for them to know that there are programs to help people in the community, not just Hispanics, also Anglo-Saxons, Chinese. But it's **good to have more information** about you so that the program grows and appears on television to announce it? Because honestly, I think they must have many resources and a lot of food that goes to waste, and honestly, that's a waste. So, we **need to inform the community** so they can also benefit. Maybe with announcements too. **Putting up announcements in Latin stores, Chinese stores**. I don’t know. **Distributing flyers** that say: will have a meeting, and if you come, we will give you some assistance, whatever you need.” (ID 17, Male, Spanish, Married, Has kids, 50) I think if they (2-1-1) are able to **give people a very set direction for that moment**, it will help a lot. Providing the resources is great, and I love that they can text it, that makes it so convenient. But if they are able to **give someone a very actionable thing in that moment**, that's more than just start at the top of the list and work your way down. I think it would help a lot of people who are in those worst situations than myself, where maybe they call the first one and it doesn’t work, and so they give up. Because if you are able to get access to that first resource, if that first resource is the most impactful or the most likely that it's going to work, that will help you navigate the rest of the list, then you’re in a little bit less of a desperate situation. And I like too that they so frequently will be like, yeah, you can call us anytime. We can send you this list over and over again. They’ll say at the end of the call, if nothing works out, call us back. It's really good at making you feel supported and not like you’re bothering them.” (ID 9, Female, English, Divorced, No kids, 29) They need to not make people feel like they’re **not entitled to the food**.” (ID 18, Female, English, Married, No kids, 66)

#### Improve emergency food provision system

Callers suggested allowing more frequent pantry visits, upgrading the quality of food distributed, ensuring it is not expired and aligns with recipients' cultural and religious needs, ensuring food boxes contain fresh, usable items that can be combined into full meals, making food resources available at more convenient times when pantries are not overly crowded, and providing transportation options or mobile food distribution (eg, food trucks or pop-up pantries) to reach people in areas without nearby pantries. Some callers stated that you should be able to access food resources, such as food pantries, more frequently. Many callers also complained about the quality of food resources, with some callers stating they had received food they could not eat because it had expired (see Table 3).

#### Enhance informational support

Callers also suggested simplifying how people find and use resources by offering clear, actionable directions and multilingual outreach through trusted community channels, providing reminders, texting, and supportive follow-up so families feel informed, welcomed, and not stigmatized when seeking help (see Table 3).

## Discussion

As one of the largest social service providers in the U.S., the 2-1-1 system plays a critical role in addressing the unmet food needs of millions of Americans every year. With recent proposed cuts to federal food programs,^20^ 2-1-1 will likely play an increasingly important role in the U.S. food system as more people will be seeking assistance for unmet food needs. In fact, during the recent government shutdown, when SNAP benefits for the month of November were delayed, food-related calls to the 2-1-1 system quadrupled, indicating the reach of this system to people with unmet food needs.^21^ Therefore, in this paper, we explored the FI experiences of 2-1-1 food-related callers to understand who the callers are and why they ended up calling 2-1-1, and sought their suggested strategies to reduce their FI. Interviews revealed that access to high-quality food through pantries and actionable information are critical needs. Addressing them may be the most cost-effective strategy with a potential for significant impact.

We found that almost all food-related callers were at risk for FI at the time of the call based on a validated measure of FI. This finding affirms the validity of food-related calls in terms of capturing true events of FI. Prior research also shows that the 2-1-1 system is reactive to known outbreaks of FI: several states observed spikes in food-related calls, both at the state and ZIP Code levels, immediately after known outbreaks of FI caused by the COVID-19 pandemic, natural disasters, or a government shutdown.^3,21-23^ As validity is one of the major attributes of any public health surveillance system,^24^ future research should explore the feasibility of using the 2-1-1 system as a real-time surveillance system for FI. As 2-1-1 callers requested improving emergency food providers' services, in terms of both quantity and quality, a real-time surveillance system for FI, informed by 2-1-1 food-related calls, can inform resource allocations among emergency food providers to meet the demand promptly. Such a surveillance system can have a significant impact, as food pantries have become a major source of food for millions of Americans in recent years.^25^

In our sample, many participants reported newly experiencing FI, suggesting that 2-1-1 calls can serve as an early warning sign of FI outbreaks. This finding also highlights the importance of providing timely information and maintaining ongoing contact with these callers to ensure their current issues do not become chronic. To support this, the 2-1-1 agencies could offer follow-up calls or develop a “starter kit” that guides newly-in-need callers through available resources.

We also found that many individuals called 2-1-1 to get help with food as they were ineligible for federal nutrition assistance programs due to their slightly higher income or immigration status. The development of a surveillance system can also be helpful here to identify areas (eg, county, state) with increased call volumes to advocate for policy changes in federal nutrition assistance programs' eligibility criteria. This was also one of the suggested strategies by callers: Strengthen and adapt federal nutrition assistance programs. However, recent funding cuts to federal nutrition assistance programs may make addressing this suggestion more challenging.^20^ Also, although not the focus of this qualitative study, it is important to note that annual adjustments to the federal poverty level, which is used to determine eligibility for federal nutrition assistance programs, generally do not keep pace with inflation in key household expenses such as food and housing. Therefore, many callers may experience rising cost-of-living pressures long before these economic shifts are reflected in the federal poverty level.

Callers were also disconnected from available food resources for which they could be eligible. For instance, among SNAP non-participants, 60% could be eligible for SNAP, based on their self-reported income and the SNAP income eligibility limit in Utah. While the 2-1-1 system in Utah assesses SNAP eligibility of callers based on their income and refers potentially eligible callers to non-profits that assist people with applying for SNAP, internal Utah 2-1-1 data show that only around 20% of referred callers end up accessing those non-profits, partly due to their limited staff and support. In other areas, such as San Diego, California, the 2-1-1 agency can complete the SNAP application during the phone call by collecting callers' signatures on the application and submitting it,^26^ which is not allowed in Utah. Therefore, allowing 2-1-1 agencies to collect callers' electronic signatures on their SNAP applications can facilitate callers' access to SNAP. Until this becomes a norm across the U.S., the 2-1-1 agencies can provide more information about the application process. While training and providing human navigators for these time-consuming tasks are costly, these agencies can use AI-assisted chatbots to provide additional information, or they can develop multilingual, low-literacy materials to share with callers through text messages.

As FI, or the ability to afford food, is a key barrier to healthy eating,^27^ 63% of callers were experiencing nutrition insecurity. As a result, diet-related health conditions were prevalent in our sample and even higher than the reported rates at the state level in three cases out of four: 23% had diabetes (Utah rate: 8.7% in 2021^28^), 23% had heart disease (Utah rate: 6.2% in 2023^29^), 33% had hypertension (Utah rate: 27.3% in 2021^30^), and 30% had obesity (Utah rate: 33.1% in 2023^31^). This finding may suggest that the 2-1-1 system could provide an opportunity for nutrition education to manage diet-related chronic diseases. Providing such information to callers can prevent the catastrophic downstream impacts of FI. While the 2-1-1 agencies might not have the capacity to train navigators to provide such information, they could train AI-assisted chatbots to provide such information or partner with relevant organizations to connect with callers.

Finally, callers were experiencing non-income-related barriers in accessing food, such as cultural barriers, stigma, discrimination, and transportation barriers, suggesting that the 2-1-1 system provides an opportunity to address those barriers at the time of call. For instance, the 2-1-1 agencies could provide anti-stigma information or connect people to food pantries with available culturally diverse foods. The latter requires a close collaboration with local food banks to map available cultural foods.

## Conclusion

In this study, we explored the FI experiences of 2-1-1 food-related callers and their suggested strategies to address FI. Callers emphasized the need for strengthening and adapting federal nutrition assistance programs, improving the emergency food provision system, and enhancing informational support. Given the recent cuts to federal nutrition assistance policies, we recommend using 2-1-1 data to improve the ability of emergency food providers to meet the local demand for food, and suggest that 2-1-1 agencies provide clear and accessible information to individuals experiencing FI. Interviews revealed that access to high-quality food through pantries and actionable information are critical needs. Addressing them may be the most cost-effective strategy with a potential for significant impact.

## Supplementary Material

qxag063_Supplementary_Data
