# Supplementary material for: From calls to action: leveraging the 2-1-1 system to intervene on food insecurity
Source: Health Aff Sch. 2026 Mar 14;4(3):qxag063. doi: 10.1093/haschl/qxag063 (PMC13032864; doi:10.1093/haschl/qxag063)
Supplement: qxag063_Supplementary_Data [file qxag063_supplementary_data.zip › Supplementary Materials-v1 (3).pdf]

Title: From Calls to Action: Leveraging the 2-1-1 System to Intervene on Food Insecurity.

## Supporting Material 1 - Consolidated Criteria for Reporting Qualitative Research (COREQ): 32-Item Checklist

Developed from: Tong A, Sainsbury P, Craig J. Consolidated criteria for reporting qualitative research (COREQ): a 32-item checklist for interviews and focus groups. *International journal for quality in health care*. 2007;19(6):349-357

**Table 1: The 32-item checklist based on the Consolidated Criteria for Reporting Qualitative Research (COREQ)**

| **No. Item** | **Guide questions/description** | **Reported on Page #** |
|---|---|---|
| **Domain 1: Research team and reflexivity** | | |
| | ***Personal Characteristics*** | |
| **1. Interviewer/facilitator** | Which authors conducted the interview? | Page 7 – Data Collection. |
| **2. Credentials** | What were the researcher's credentials? | See the Title Page |
| **3. Occupation** | What was their occupation at the time of the study? | See the Title Page |
| **4. Gender** | Was the researcher male or female? | Male: First, fifth, seventh, and ninth authors.<br>Female: Other authors. |
| **5. Experience and training** | What experience or training did the researcher have? | Page 7 – Data Collection. |
| ***Relationship with participants*** | | |
| **6. Relationship established** | Was a relationship established prior to study commencement? | Only with the Utah 2-1-1 agency. |
| **7. Participant knowledge of the interviewer** | What did the participants know about the researcher? | Nothing |
| **8. Interviewer characteristics** | What characteristics were reported about the interviewer? e.g. Bias, assumptions, reasons and interests in the research topic | We only let them know that the interviewers can speak English and Spanish. |
| **Domain 2: study design** | | |
| | **Theoretical framework** | |
| **9. Methodological orientation and Theory** | What methodological orientation was stated to underpin the study? e.g. grounded theory, discourse analysis, ethnography, phenomenology, content analysis | Page 10 – Data Analysis section |
| | **Participant selection** | |
| **10. Sampling** | How were participants selected? e.g. purposive, convenience, consecutive, snowball | Page 7 – Recruitment section |
| **11. Method of approach** | How were participants approached? e.g. face-to-face, telephone, mail, email | Page 7 – Recruitment section |
| **12. Sample size** | How many participants were in the study? | Page 7 – Data Collection section |
| **13. Non-participation** | How many people refused to participate or dropped out? Reasons? | Page 7 – Recruitment section |
| | **Setting** | |
| **14. Setting of data collection** | Where was the data collected? e.g. home, clinic, workplace | Page 7 – Data Collection section |
| **15. Presence of non-participants** | Was anyone else present besides the participants and researchers? | No. |
| **16. Description of sample** | What are the important characteristics of the sample? e.g. demographic data, date | Pages 12-13 – Results section |
| | **Data collection** | |

| | | |
|---|---|---|
| **17. Interview guide** | Were questions, prompts, guides provided by the authors? Was it pilot tested? | The authors developed the interview questions and guides. It was pilot tested by the authors. |
| **18. Repeat interviews** | Were repeat interviews carried out? If yes, how many? | No. |
| **19. Audio/visual recording** | Did the research use audio or visual recording to collect the data? | Audio recording. |
| **20. Field notes** | Were field notes made during and/or after the interview? | No. |
| **21. Duration** | What was the duration of the interviews? | Page 7 – Data Collection section |
| **22. Data saturation** | Was data saturation discussed? | Page 10 – Data Analysis section |
| **23. Transcripts returned** | Were transcripts returned to participants for comment and/or correction? | No. |
| **Domain 3: analysis and findings** | | |
| **Data analysis** | | |
| **24. Number of data coders** | How many data coders coded the data? | Page 10 – Data Analysis section |
| **25. Description of the coding tree** | Did authors provide a description of the coding tree? | No. |
| **26. Derivation of themes** | Were themes identified in advance or derived from the data? | From the data. |
| **27. Software** | What software, if applicable, was used to manage the data? | Page 10 – Data Analysis section. Dedoose |
| **28. Participant checking** | Did participants provide feedback on the findings? | No. |
| **Reporting** | | |
| **29. Quotations presented** | Were participant quotations presented to illustrate the themes/findings? Was each quotation identified? e.g. participant number | Tables 2-3 |
| **30. Data and findings consistent** | Was there consistency between the data presented and the findings? | Yes. |
| **31. Clarity of major themes** | Were major themes clearly presented in the findings? | Yes. Tables 2-3 |
| **32. Clarity of minor themes** | Is there a description of diverse cases or discussion of minor themes? | No. |
